# Prevalence and determinants of early onset neonatal sepsis at two selected public referral hospitals in the Northwest Ethiopia: a cross-sectional study

**DOI:** 10.1186/s12887-022-03824-y

**Published:** 2023-01-05

**Authors:** Tadesse Yirga Akalu, Yared Asmare Aynalem, Wondimeneh Shibabaw Shiferaw, Melaku Desta, Haile Amha, Dejen Getaneh, Bayachew Asmare, Yoseph Merkeb Alamneh

**Affiliations:** 1 College of Health Science, Debre Markos University, P.O. Box 269, Debre Markos, Ethiopia; 2 College of Health Science, Debre Berihan University, Debre Berihan, Ethiopia; 3 College of Health Science, Debre Tabor University, Debre Tabor, Ethiopia; 4 School of Medicine, Debre Markos University, Debre Markos, Ethiopia

**Keywords:** Early-onset sepsis, Ethiopia, Neonate, Prevalence, Determinants

## Abstract

**Introduction:**

Globally, neonatal mortality is decreasing, and road maps such as the Early Newborn Action Plan set ambitious targets for 2030. Despite this, deaths in the first weeks of life continue to rise as a percentage of total child mortality. Neonatal sepsis with early onset continues to be a significant cause of death and illness. The majority of sepsis-related deaths occur in developing nations, where the prevalence and causes of newborn sepsis are yet unknown. As a result, the goal of this study was to determine the prevalence of early-onset sepsis and identify determinant factors.

**Methods:**

A cross-sectional study was conducted on 368 study participants in referral hospitals of East and West Gojjam Zones from March 1^st^ to April 30^th^, 2019. Study participants were selected at random using lottery method. Face-to-face interviews with index mothers for maternal variables and neonatal record review for neonatal variables were used to collect data using a structured pretested questionnaire. Data were entered into Epidata 3.1 and then exported to STATA/SE software version 14. Finally, the logistic regression model was used for analysis. Statistical significance was declared at *P* < 0.05 after multivariable logistic regression.

**Results:**

A total of 368 newborns and their index mothers took part in this study. The mean age of the newborns was 4.69 days (± 1.93SD). Early-onset neonatal sepsis was seen in 34% of the babies. Nulliparity (AOR: 3.3, 95% CI: 1.1–9.5), duration of labor > 18 h after rupture of membranes (AOR: 11.3, 95% CI: 3.0—41.8), gestational age of 32–37 weeks (AOR: 3.2, 95% CI: 1.2—8.5), and neonates who require resuscitation at birth (AOR: 4, 95% CI: 1.4 -11.8) were all found to be significantly associated with early-onset neonatal sepsis.

**Conclusion and recommendation:**

Early-onset neonatal sepsis was found to be high in this study. Early-onset neonatal sepsis was found to be associated with maternal, obstetric, and neonatal variables. Comprehensive prevention strategies that target the identified risk factors should be implemented right away.

## Introduction

A neonate, often known as a newborn infant, is a baby who is born within the first 28 days of life [1]. The phrase "neonatal sepsis" refers to the systemic response to infection in newborns within the first four weeks following delivery [2]. It is a clinical pattern that develops as a result of microbial blood infection in the first month of life [3]. Neonatal sepsis is defined as early-onset sepsis (if the onset of clinical features occurs between birth and 7 days) or late-onset neonatal sepsis (LONS) if the onset of clinical features occurs between 8 and 28 days after birth [4, 5]. Early onset neonatal sepsis (EONS) has been defined in a variety of ways depending on the age of commencement, with bacteremia occurring as early as 7 days after birth [3]. Early onset neonatal sepsis (EONS) develops vertically from the mother and presents soon after birth [6]. EONS are caused by bacteria that infect the maternal genitourinary system, contaminating the amniotic fluid, placenta, cervix, and vaginal canal. When the amniotic membranes burst before delivery, the pathogen may ascend, causing an intra-amniotic infection. As a result, the infection can be acquired by the infant either in pregnancy or at delivery [7].

Maternal and neonatal risk factors for EONS include maternal age of 35 or less than 20 years, preterm, parity, cesarean delivery, urinary tract infection in the third trimester of pregnancy, and neonate sex, meconium stained amniotic fluid, birth asphyxia, and birth weight [8, 9]. Procedures that change the amniotic cavity during pregnancy, such as cervical cerclage and amniocentesis, can raise the risk of intra-amniotic infection and neonatal sepsis [10]. Prematurity, low birth weight, congenital malformations, complex or instrument-assisted delivery, [3]and low APGAR scores (score of 6 at 5 min) are all associated with EONS in addition to the mother's variables [11, 12]. Premature neonatal immune system immaturity, particularly low immunoglobulin G (IgG) levels due to diminished maternal IgG trans placental transfer, increases the risk of sepsis in preterm newborns [13, 14].

Neonatal death is still the leading cause of death among children under the age of five [15]. Prematurity, asphyxia, and sepsis account for 87% of neonatal deaths worldwide [16]. According to World Health Organization, nearly 45% of under-five deaths accounts for neonatal death, with 75% occurring in the first seven days of life, and sepsis being the second most common cause**.** [17]. Neonatal death rates from EONS range from 16.7 to 40% [6, 18, 19]. Despite significant advancements in neonatal care, 40% of infants die with sepsis or suffer with neurodevelopmental impairment as a result of a lack of laboratory reagents to detect early-onset neonatal sepsis [20].

Because of greater Group B Streptococci treatment during pregnancy, the proportion of EONS relative to late onset sepsis (LOS) has been quickly reducing in high-income countries (HIC) [21, 22]. Despite EONS can be treated and weakened quickly, it can also cause neonatal death in a matter of hours or days [23].Early onset neonatal sepsis is a common and serious problem for neonates, particularly preterm newborns [24].

The primary target of the national newborn and child survival plan is to reduce under-five mortality from 64 to 29 per 1000 children, infant mortality from 44 to 20 per 1000 children, and neonatal death from 28 to 11 per 1000 children by 2020 [25]. Identifying risk factors and putting in place core interventions are the keys towards preventing 415,688 and 210,234 deaths in children under the age of five and neonates, respectively [17]. As a result, quantifying the true number of cases of early-onset newborn sepsis in underdeveloped nations is extremely challenging [26]. A recent systematic review and meta-analysis in Ethiopia showed the prevalence of EONS was 75.44% and further studies were recommended [27]. Consequently, data on early onset infant sepsis is limited in the research area, which has the greatest rate of early neonatal death. As a result, the primary goal of this study was to quantify the prevalence of early-onset newborn sepsis in Northwest Ethiopia and identify associated factors. Knowledge of determinant factors related to early onset neonatal sepsis helps the clinician for early recognition and lowering death and illness.

## Materials and methods

### Study area and period

The study was done from March 1^st^ to April 30, 2019, in public referral hospitals in the East and West Gojjam zones of Ethiopia's Amhara Region. There are only two public referral hospitals in the two zones, and both were included in the study since they provide inpatient neonatal treatment. Debre Markos Referral Hospital is located in Debre Markos, the town of East Gojjam Administrative Zone, while Felege Hiwot Referral Hospital is located in Bahir Dar, the town of Amhara regional state. Both are located in Northwest Ethiopia. According to information gathered from these hospitals' administrative offices, they offer a variety of services in the outpatient, inpatient, and operating room theatre departments. Debre Markos Referral Hospital [28] and Felege Hiwot Referral Hospital [29] serve a catchment area of around 3.5 million and 5 million people, respectively.

### Study design and population

#### Study design and population

Data were collected from March 1^st^ to April 30^th^, 2019, among neonates admitted to neonatal intensive care units using a hospital-based cross-sectional study design. The source population included all neonates who received care as inpatients or outpatients in the specified public referral hospital. During the data collecting period, all neonates admitted to neonatal intensive care units (NICUs) were included as study population. The study included neonates under the age of seven days who were admitted in the two public referral hospitals. Neonates with early discharge, incomplete charts, and died on arrival were all excluded from the study.

### Sample size determination

The single population proportion formula was used to calculate the sample size. Given the prevalence of early onset sepsis, which is estimated to be 65% [12]. The level of confidence is 95%, while the margin of error is 5%. After calculating the sample size, a 5% non-response rate was added, yielding a total sample size of 368.

$$\begin{array}{cc}\mathrm{n}= \frac{{\left(\frac{\mathrm{Za}}{2}\right)}^{2}(\mathrm{P})(1-\mathrm{P})}{{(0.05)}^{2}}& \mathrm{n}=\frac{{(1.96)}^{2}(0.65)(0.35)}{{(0.05)}^{2}}=350\end{array}$$

where: n – initial sample size

- Z – standard normal value at 95% CI which is 1.96
- P – Prevalence of early onset sepsis 65%
- d – Possible margin of error tolerated which is 5%

### Sampling techniques and procedure

The study participants were selected using a simple random sampling technique using the medical registration numbers of neonates admitted to neonatal intensive care units. For the NICUs at the two hospitals, the samples were distributed proportionally using the probability proportional to size (PPS) allocation technique (215 samples from Felege Hiwot Referral Hospital and 153 samples from Debre Markos Referral Hospital).

### Operational definition

Early onset neonatal sepsis: was defined as presence of at least one of [difficulty feeding, history of convulsions, movement only when stimulated, respiratory rate of 60 or more breaths per min, severe chest retractions, or a temperature of 37.5 °C or higher or 35.5 °C or lower, Cyanosis, grunting and change in level of activity] with in the first seven days after birth [30]. Presence of any one of clinical signs and symptoms predict severe infection (based on an expert pediatrician’s assessment) and was associated with a sensitivity and specificity of 85% and 75% [31].

### Data collection tool and quality control

The questionnaire was written in English and then translated into the local language. An impartial translator checked for consistency by re-translating it into English. Four diploma nurses and two BSc degree nurses were recruited as data collectors and supervisors respectively. Before the actual data collection, the data collectors and supervisors were briefed for two days on data collection procedures and study objectives. In Finote Selam Hospital, a pretest was conducted using 5% of the total sample size, which was not included in the actual sampling, and necessary tool adjustments were made. Data was collected through a pretested questionnaire administered by an interviewer. Index mothers were interviewed and neonatal records were reviewed using checklists for neonatal characteristics such as the APGAR (Activity, Pulse, Grimace, Appearance, and Respiration) score. Supervisors and investigators oversaw the data gathering technique on a daily basis to ensure the quality of the data. A review was conducted to ensure that the questionnaire was complete, and corrections were made. Prior to data entry, each questionnaire and data sheet were double-checked.

### Data processing and analysis

The data were entered into the EPI-data Version 3.1 software. The data that were entered was checked and cleaned. Then, it was exported to STATA version 14 for analysis. To describe the research variables in relation to the population, descriptive statistics such as frequency, proportions, and percentages were generated and displayed in tables and graphs. To see if there were any relationships between the dependent and explanatory variables, bivariable and multivariable logistic regressions were used. To determine the relationship between the two variables, the odds ratio and *p*-value were calculated. To adjust for probable confounders, variables with a *p*-value ≤ 0.25 were entered into a multivariable logistic regression model. Finally, statistical significance was declared at a *P*-value of ≤ 0.05 and 95% confidence interval.

## Results

### Socio-demographic characteristics of study participants

A total of 368 newborns and their index mothers took part in this study, with a 100% response rate. The mean age of neonates was 4.69 days with (± 1.93 SD), while the mean age of index mothers was 29.5 years with (± 7 SD). More over half of the index mothers (232) were between the ages of 20 and 34, and the majority of the study participants (84%) were married. When it came to where they lived, the majority (263, or 71.5%) were from urban centers. In terms of household income, 179 (48.6%) of study participants had a low socioeconomic level. In terms of gender, female neonates comprised 199 (54.08%) of the total, while male neonates comprised 169 (45.92%). (Table 1).

**Table 1:** Socio-demographic characteristics of neonates and their index mothers admitted at two selected Public Referral Hospitals in the Northwest Ethiopia: 2019

Variables	Categories	frequency	percentage
Maternal age	< 20	28	7.6%
	20–34	232	63.0%
	> = 35	108	29.4%
Marital status	Single	32	8.7%
	Widow	26	7.1%
	Married	310	84.2%
Maternal religion	Orthodox	231	62.8%
	Muslim	92	25.0%
	Protestant	45	12.2%
Maternal occupation	Housewife	153	41.6%
	Civil servants	94	25.5%
	private employed	48	13.0%
	Business woman	73	19.8%
Neonatal sex	Male	169	45.9%
	Female	199	54.1%

### Gynecologic and obstetric characteristics of the mother

**Fig. 1:**
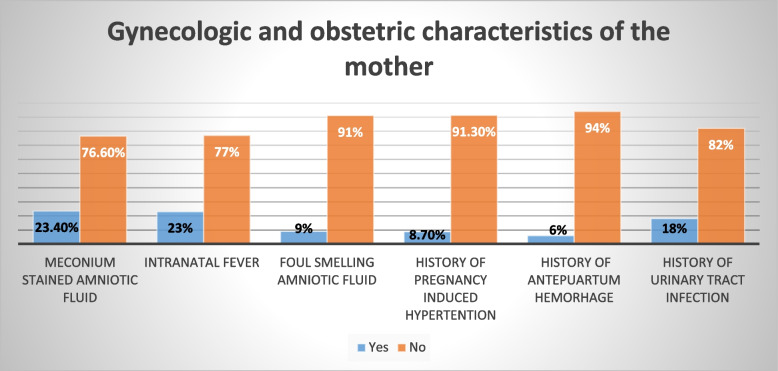
Gynecologic and obstetric characteristics of index mothers for the study on prevalence and determinants of Early Onset Neonatal Sepsis at two selected public referral Hospitals in the Northwest Ethiopia: 2019

In terms of antenatal care (ANC), the majority of women 305 (82.88%) received ANC during their current pregnancy, with 238 (64.8%) receiving complete ANC. In terms of method of birth, the proportion of newborns delivered spontaneously was greater 208 (56.5%) than the number of neonates born via caesarian Sect. 40(10.9%) and instrumental 120(32.6%). Similarly, 351 (95.4%) of infants were attended by health care professionals. The proportion of neonates born to mothers whose labor lasted less than 12 h following rupture of membranes was roughly half of the study participants 179 (48.64%). However, 54 (14.67%) of women had a labor that lasted more than 18 h following the rupture of the membrane. Twenty three present of index mothers had intranatal fever. But, most of the index mothers (77%) had no history of intranatal fever besides Majority of mothers (91%, 94%) had no history of foul smelling amniotic fluid and antepuartum hemorrhage respectively. Majority of index mothers, (82%, and 91.3%) had no history of urinary tract infection (UTI) and pregnancy induced hypertension (PIH) respectively during their pregnancy (Fig. 1).

### Birth characteristics of neonatal variables

In terms of gestational age, the majority of the study participants 253 (68.7%) were born between 37 and 42 weeks after the last normal menstrual period. Similarly, the majority of the study participants' first and fifth minute APGAR scores were above seven, with 265 (72.01%) and 293 (79.62%) respectively. In terms of neonatal weight, more than half 228 (62.0%) were born in the normal birth weight range (2500–4000 g), although a small percentage of neonates were either overweight (> 4000 g) or very low birth weight (1500 g), accounting for 14(3.8%) and 13(3.5%), respectively. Similarly, the majority of the study participants 250 (67.9%) cried right after birth (Table 2).

**Table 2:** Birth characteristics of neonates who were admitted at two selected Public Referral Hospitals in the Northwest Ethiopia: 2019

Variables	Categories	frequency	Percentage
Gestational age of the neonate at birth	> 42 weeks	14	3.8%
	37–42 weeks	253	68.8%
	32–37 weeks	88	23.9%
	< 32 weeks	13	3.5%
First minute APGAR score	< 7	93	25.3%
	> 7	265	72.0%
Fifth minute APGAR score	< 7	65	17.7%
	> 7	293	79.6%
Birth weight	> 4000 g	14	3.8%
	2500–4000 g	228	62.0%
	1500-2500 g	113	30.7%
	< 1500 g	13	3.5%
Immediate cry at birth	Yes	250	67.9%
	No	118	32.1%
Resuscitated at birth	Yes	96	26.1%
	No	272	73.9%

### Associated factors of early onset neonatal sepsis

This study was intended to determine the prevalence of early onset neonatal sepsis and its determinant factors. Early onset neonatal sepsis was found in 34% of newborns admitted during the study period. In the multivariable logistic regression, all variables with a *P*-value of less than 0.25 in bivariable analysis (Maternal age, Maternal age, Maternal parity, Duration of labor after rupture of membranes, Number digital of per-vaginal examination, Gestational age, Birth weight, Resuscitation at birth) were adjusted for nulliparity, duration of membrane rupture, gestational age, and resuscitation at birth were all found to be significantly associated with the occurrence of early onset neonatal sepsis in the adjusted multivariable logistic regression.

Maternal parity was found to be a significant risk factor for neonatal sepsis with early onset neonatal sepsis. In particular, neonates born from nulliparous mothers were three times (AOR: 3.3, 95% CI: 1.1- 9.5) more likely than those born to para one mothers to develop early onset sepsis.

The duration of labor following membrane rupture was revealed to be a major determinant in early onset neonatal sepsis. Specifically, neonates born after a labor lasting more than 18 h (AOR: 11.3, 95% confidence CI: 3.0—41.8) were eleven times (AOR: 11.3, 95% CI: 3.0—41.8) more likely to develop early onset sepsis than neonates born after a labor lasting less than 12 h. Neonatal gestational age was also found to be a major predictor of neonatal sepsis with early onset. Early onset sepsis was three times more common in neonates born between 32 and 37 weeks gestational age (AOR: 3.2, 95% CI: 1.2—8.5) than in neonates born between 37 and 42 weeks gestational age. Furthermore, resuscitation at birth was found to be an independent predictor of neonatal sepsis with early onset. When compared to neonates who were not resuscitated at birth, neonates who were resuscitated at birth were approximately four times more likely to develop early onset neonatal sepsis (AOR: 4, 95% CI: 1.4 -11.8) (Table 3).

**Table 3:** Bivariable and multivariable logistic regression showing the association between EONS and the different variables in neonates admitted at two selected Public Referral Hospitals in the Northwest Ethiopia: 2019

Variables	Category	Early onset sepsis		COR with 95%CI	AOR with 95%CI
		Yes	No		
		Count (Percent)	Count (percent)		
Maternal age	< 20	20 (16)	8 (3.3)	5.3(2.2–12.7)	
	20–34	74 (59.2)	158 (65)	1	
	> 35	31 (24.8)	77 (31.7)	0.8(0.5–1.4)	
Maternal parity	Nulli-para	43 (34.4)	40 (16.5)	2.1(1.1–3.7)	3.3 (1.1- 9.5)
	Para-one	48 (38.4)	95 (39.1)	1	1
	Multi-para	34 (27.2)	108 (44.4)	0.6(0.4–1.0)	
Duration of labor after rupture of membrane	< 12 h	27 (21.6)	152 (62.6)	0.3(0.2 -0.5)	
	12-18 h	52 (41.6)	83 (34.2)	1	1
	> 18 h	46 (36.8)	8 (3.3)	9(4.0 -21.0)	11.3(3.0 -41.8)
Number digital of per-vaginal examination	< 3	24 (19.2)	134 (55.0)	1	
	> 3	101 (80.8)	109 (45.0)	5(3.1- 8.63)	
Gestational age	> 42 weeks	4 (3.2)	10 (4.1)	1.5(0.4 -4.8)	
	37–42 weeks	54 (43.2)	199 (81.9)	1	1
	32–37 weeks	60 (48.0)	28 (11.5)	7.8(4.6–13.6)	3.2(1.2—8.5)
	< 32 weeks	7 (5.6)	6 (2.5)	4.3(1.4–13.3)	
Birth weight	> 4000 g	5 (4.00	9 (3.7)	2(0.7–6.5)	
	2500–4000 g	48 (38.4)	180 (74.1)	1	
	1500-2500 g	64 (51.2)	49 (20.2)	4.8(3.0 -8.0)	
	< 1500 g	8 (6.4)	5 (2.1)	6(1.8—19.2)	
Resuscitation at birth	yes	70 (56.0)	26 (10.7)	10.6(6.2–8.2)	4(1.4 -11.8)
	No	55 (44.0)	217 (89.3)	1	1

## Discussion

The goal of this study was to determine the prevalence of early onset neonatal sepsis (EONS) and identify possible determinant factors in neonates admitted to public referral hospitals in Northwest Ethiopia. According to this finding, the prevalence of early onset neonatal sepsis was 34%, and nulliparity, duration of membrane rupture, gestational age, and resuscitation at birth were the significant independent predictors.

Early onset sepsis was found to be prevalent in 34% of research participants referred to neonatal intensive care units at the two selected referral hospitals. This conclusion was consistent with a study conducted in Dil Chora Referral Hospital, Eastern, Ethiopia [32]and South Sinai, Egypt [33] which found that the prevalence of early onset neonatal sepsis in neonatal intensive care units was 40.5% and 31.8% respectively. Early onset neonatal sepsis (EONS) was found to be higher than studies conducted in Indonesia 26.6% [34] and in India 20.9% [35], in hospitals of Wolaita Sodo Town 26.9% [36]. The primary cause of the disparity could be differences in sociodemographic factors. Women in these developed countries are thought to have a higher level of awareness and knowledge. Women in Ethiopia, on the other hand, are considered ineligible for school and financially unable to care for their newborns. The second likely explanation is that diagnostic criteria for cases of EONS varied. For example, in our study, we employed just clinical signs suggestive of sepsis, which overestimates EONS, whereas studies in developed countries, culture positive results were used to identify EONS. Other possible factors were no standardized policy for screening for infections in asymptomatic pregnant women and poor antenatal care which could result in insufficient time for maternal antibiotic coverage prior to or during labour. This finding was lower than a systematic review and meta-analysis in Ethiopia 75.4% (95% CI: 68.3, 82.6) [27], Gondar 59.6% [37], in Bishoftu General Hospital, Neonatal Intensive Care Unit, Debrezeit-Ethiopia 81.4% [13], in a tertiary center in Kathmandu, Nepal 91.39% [38] Public Hospitals of Hawassa City Administration, Southern Ethiopia,80.9% [39]. This variation could be due to unique cultural features of the population, local obstetrics and neonatal practices, socioeconomic and sexual practice, hygiene, and nutritional differences over settings [33] as well as due to clinical features for sepsis identification, study methodology, and sample size differences were observed among studies.

The occurrence of EONS was shown to be significantly associated with gestational age in this study. When compared to term neonates, neonates born less than 37 weeks were three times more likely to develop early onset sepsis. This finding was supported by studies in Indonesia [40], Mexico [41], India [42], South Africa [43], Pakistan [44], Ghana [45], Nepal [46] and China [47]. This association could be related to premature newborns' undeveloped immune systems and malfunctioning neutrophils. Furthermore, premature newborns lack complement proteins, making them prone to sepsis ascending [48]. Furthermore, premature neonates have insufficient immunoglobulin G (IgG), making them susceptible to sepsis from pathogenic microorganisms [49].

Early onset newborn sepsis was found to be significantly associated with the length of labor following membrane rupture. In particular, neonates born from women who had a labor after rupture of membrane that lasted more than 18 h were eleven times more likely to suffer with early onset sepsis than those born from women who had a labor after rupture of membrane that lasted less than 18 h. This finding was consistent with research conducted in the United States [50], Thailand [51], Tanzania [52] and India [42] that found labor time to be an independent determinant factor for early onset sepsis. Membrane rupture that lasts a long time (PROM) before delivery was associated with Chorioamnionitis which poses direct fetal risks from vertical transmission or ascending infection from vaginal flora due to the loss of a barrier, it is possible that PROM could be an independent risk factor for early onset neonatal sepsis.

Furthermore, maternal parity was found to be an independent predictor of with early onset neonatal sepsis. When compared to neonates delivered to para-one mothers, neonates from nulliparous women were three times more likely to develop early onset neonatal sepsis. A study conducted in Ghana [45], India [53], and South Africa [43] supports this finding. Null parity is commonly accompanied with a number of sepsis related factors, including prolonged labor and repeated per-vaginal digital examinations. Early onset neonatal sepsis was also observed to be associated with resuscitation at birth. When compared to neonates who were not resuscitated at birth, neonates who were resuscitated at birth were four times more likely to have early-onset sepsis. This finding was supported by research conducted in Tanzania [54], Ghana, [45] and the United States [50]. Poor practices and non-compliance with guidelines by health professionals during the process may expose the neonate to a higher risk of sepsis. These findings could be due to the fact that, if procedure of resuscitation is done forcefully, it may cause laceration to the susceptible and easily breakable mucous membrane of the neonate and serve as a route of entry for pathogens from unsterile equipment [5]. It’s going to also lead microbes into the lower air way of the newborn with an immature immune system. This is often due to the lumen of airways of the neonate is too narrow, and respiratory secretions are copious compared to older children which could predispose to easily destruction of smaller air sacs and leads to sepsis. In conclusion, the outcomes of this study reveal that the prevalence of early onset newborn sepsis was found to be considerably higher at the two public referral hospitals. In addition, maternal obstetric parameters such as maternal parity, duration of labor after membrane rupture, and neonatal variables such as gestational age of the neonate and neonatal resuscitation at birth were revealed to be risk factors for early onset neonatal sepsis. Comprehensive risk-reduction methods that target the identified determinants should be improved. As a result, health care providers working in neonatal intensive care units should follow guidelines when performing invasive procedures, improve maternal education on determinants such as prolonged rupture of membrane (PROM), and incorporate routine neonatal sepsis screening into neonatal and maternal care. To avoid early-onset newborn sepsis, neonates who have been resuscitated at delivery and those born to nulliparous should get special attention.

### Strength and limitations of the study

This research has a number of advantages. First, we used primary data, with the exception of certain neonatal variables, to reduce the number of missing values. Second, it was carried out relatively over a wider research region. But, this study is not unique, because participant memory and self-report nature of determinant factors are potential limitations that could cause bias. Furthermore, sepsis cases were not identified based on culture-confirmed laboratory results. Neonates with signs and symptoms of sepsis may not be truly septic for culture, this could expose our findings to selection bias. Therefore, the true figure of EONS could more or less than the findings of this study for it’s based only on sign and symptoms.

## Data Availability

The data sets analyzed during the current study are available from the corresponding author upon reasonable request.

## Abbreviations

ANC: Antenatal Care
APGAR: Activity, Pulse, Grimace, Appearance, Respiration
CI: Confidence Interval
EONS: Early Onset Neonatal Sepsis
LONS: Late Onset Neonatal Sepsis
NICU: Neonatal Intensive Care Unit
AOR: Adjusted Odds Ratio
PROM: Prolonged Rupture of Membrane
